# Reply to: Available data do not rule out Ctenophora as the sister group to all other Metazoa

**DOI:** 10.1038/s41467-023-36152-5

**Published:** 2023-02-10

**Authors:** Anthony K. Redmond, Aoife McLysaght

**Affiliations:** Smurfit Institute of Genetics, Trinity College Dublin, Dublin, Ireland

**Keywords:** Molecular evolution, Phylogenetics, Phylogeny

**replying to** N. V. Whelan & K. M. Halanych *Nature Communications* 10.1038/s41467-023-36151-6 (2023)

In Redmond and McLysaght^1^ (R&M) we integrated precomputed site-heterogeneous amino acid substitution models and amino acid recoding into partitioned phylogenomics and showed that this improved model-fit and resistance to long-branch attraction (LBA; a common phylogenetic error). These better fitting modelling strategies revealed a shift in support away from Ctenophora (comb jellies), and towards Porifera (sponges), as sister to all other animals^1^. Whelan and Halanych^2^ (W&H) criticised our recoded analyses and interpretations on the placement of Porifera and Ctenophora in the animal phylogeny. Here we counter these criticisms, and bolster evidence for Porifera as sister to all other animals.

W&H claim we did not adequately consider well-accepted relationships (called ‘generally accepted topologies’ in R&M^1^) when assessing the performance of new approaches in R&M^2^. The site-heterogeneous models and recoding strategy that we employed have been tested and used elsewhere (see R&M^1^), and we deemed further validation redundant. However, we did test the ability of partitioned site-heterogeneous models and recoding to combat specific and well-characterised LBA problems in real datasets^3,4^ (see R&M^1^), finding that both improved upon standard partitioned phylogenomics^1^. Although we agree that new approaches should recover well-accepted clades, W&H’s argument against our approaches (particularly recoding), based on failure to recover Chordata (LEA[N/P] datasets^1^) and Deuterostomia (WEA17 dataset and reduced support in BEA dataset^1^), is weak. Whether deuterostomes are monophyletic is currently unresolved^5^, meaning deuterostome non-monophyly cannot convincingly cast doubt on our site-heterogeneous, recoded analyses of WEA17 (including recovery of Porifera-sister) or BEA. The LEA(N/P) datasets were not designed to assess chordate monophyly^4^, and Chordata was never recovered with strong support in any of our^1^, or W&H’s^2^, LEAN/LEAP analyses, but neither was any alternative^1^. Interestingly, despite each being expected to improve phylogenetic inference, (i) using closer-related outgroups has also disrupted Chordata in some past analyses of these datasets^4^, and (ii) partitioning-by-gene outperforms W&H’s better fitting partition scheme in recovering Chordata (W&H Fig. 1  in ref. ^2^). Conspicuously, the problematic lineages, Ambulacraria and Cephalochordata, are represented by a single species each, and improved taxon sampling resolves this issue for a related dataset^6^. Thus, these datasets appear to harbour little signal either for or against Chordata, rather than recoding causing an inference problem. Given the numerous potential lineage-specific dataset and biological biases that can arise, we do not believe that data insufficient to recover one off-target clade with available modelling strategies cannot reliably be used to assess target relationships.

**Fig. 1:**
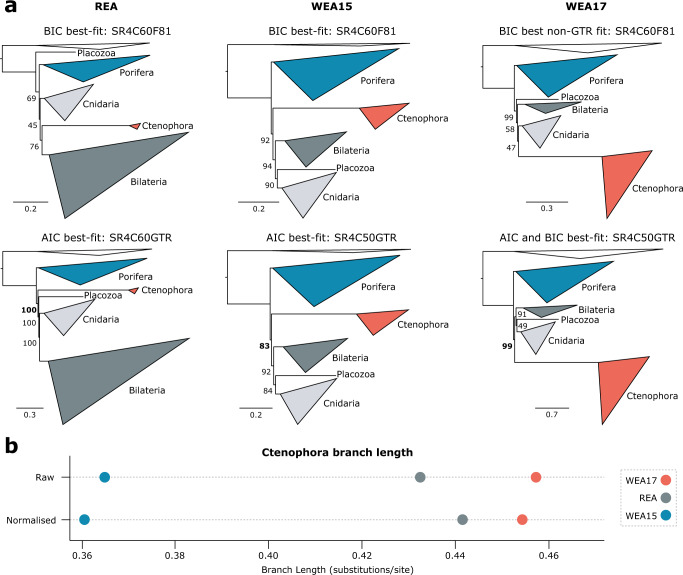
Unpartitioned recoding reanalyses and ancestral ctenophore branch lengths. **a** Reanalyses of SR4 recoded animal phylogeny datasets REA, WEA15, and WEA17 without using partitioning. Consensus trees from reanalyses with best-fitting F81-based models are shown in the top row and those from reanalyses with best-fitting GTR-based models are shown in the second row. Model-fitting results reported above each tree compared both F81 and GTR-based models (including site-homogeneous F81 and GTR). Ultrafast bootstrap support for Porifera-sister is shown in bold for the GTR-based analyses. Models are as specified in R&M^1^. **b** Length in substitutions/site of the ancestral branch of the Ctenophore clade for each dataset as analysed at the amino acid level using standard partitioned phylogenomics with site-homogeneous models (i.e., analysis level ‘L1’ of R&M^1^). ‘Raw’: branch length extracted directly from the resultant tree, ‘Normalised’: branch length divided by total tree length (and then multiplied by the mean total tree length of the three datasets for presentation in comparison to Raw).

As W&H noted, our site-heterogeneous recoded analyses showed poor resolution and inconsistencies between REA, WEA15 and WEA17, an issue they ascribe to recoding^2^. Pertinently, our site-homogeneous recoded analyses (for which GTR-based models fit best) do not suffer from this issue, and thus, in R&M we proposed that combining partitioning and recoding had reduced the number of complex alignment site patterns per partition such that simple F81 exchangeabilities fit better than GTR exchangeabilities when using site-heterogeneous models^1^. We test this here with unpartitioned SR4-recoded reanalyses of these datasets, as this might provide enough data for models that are both site-heterogeneous and GTR-based to fit best. As expected, topologies and branch supports are largely consistent with those recovered in our original RL2 analyses (R&M Fig. 3c in ref. ^1^) under the best-fitting unpartitioned site-heterogeneous F81 models (top row in Fig. 1a). However, site-heterogeneous GTR-based models fit best under AIC for all three datasets, and under BIC for WEA17. As anticipated, these GTR-based reanalyses recover highly consistent relationships between the five major animal lineages across datasets (bottom row in Fig. 1a) and with previous studies^7–9^, and all three support Porifera-sister (UFBOOT: REA = 100%, WEA15 = 83%, WEA17 = 99%; Fig. 1a). Together with Ctenophora-sister being recovered in R&M when using recoding with less well fitting site-homogeneous models^1^, these new findings clearly negate W&H’s argument that inappropriate recoding drives Porifera-sister in R&M. The issues they note are instead ascribable to using site-heterogeneous models with simple F81 (rather than GTR) exchangeabilities in partitioned analyses of recoded data, accounting for which reinforces Porifera-sister.

W&H favour the WEA17 dataset as it includes more ctenophore species^2^. While improved ingroup taxon sampling can undoubtedly improve phylogenetic inference, it is noteworthy that the extra species do not break the long branch leading to the Ctenophora clade—the very branch which is purported to cause LBA (see also^8^). This branch is longer in WEA17 than in REA or WEA15 (Fig. 1b). Regardless of whether this increased branch length is (i) an improvement resulting from better ctenophore taxon sampling and/or (ii) results from other factors (such as the different gene families, alignment sites, or errors in each dataset) it nonetheless indicates that if Ctenophora-sister arises from LBA, then it will be harder to overcome for WEA17. This is consistent with our findings in R&M^1^, where the shift (as better-fitting models are applied) towards supporting Porifera sister is recovered more slowly for WEA17 (only apparent at the partition-specific level when not recoding), and most easily for WEA15 (which has the shortest ctenophore branch). This may explain why Porifera-sister is only recovered for WEA17 when the data are recoded^1,8^. Thus, W&H’s emphasis on ctenophore sampling inadvertently prioritizes a dataset (WEA17) with increased potential for ctenophore LBA. This is a major concern given that Ctenophora-sister is far more likely than Porifera-sister to be erroneously recovered in simulations affected by LBA-inducing systematic error^10^.

W&H claim we were unfairly critical of previous studies^2^. First, we disagree that we inappropriately dismissed Hernandez and Ryan’s^11^ concerns about recoding given our above points on the recovery of Deuterostomia and Chordata in our test datasets and evidence that W&H’s issues with recoded analyses in R&M do not in fact derive from recoding. Other simulation studies have supported recoding^12^, or are at least ambivalent^13^, and as we advocated in R&M, ‘a fuller understanding of the implications of recoding is needed’^1^. Second, our claim that REA and WEA15 contain paralog contamination referenced other work^7^ and personal communication was limited to WEA17^1^, which has now been shown to support Porifera-sister without recoding when orthogroups with poor orthologous signal (i.e., inability to recover major animal lineages at the gene tree level) are excluded^14^. We concede that personal communication was less than ideal, particularly as sorting orthologs from paralogs is at least somewhat dependent on the approach employed/investigator interpretation, but our comment also referred to the relatively high percentage of missing data for ctenophores in WEA17, which is directly observable in the dataset. W&H contend that if these datasets contain paralog contamination it would invalidate the findings in R&M^2^, yet it also would invalidate the original findings favouring Ctenophora-sister, a hypothesis derived from phylogenomics. Furthermore, phylogenomic analyses with site-homogeneous models, from which the strongest evidence and support for Ctenophora-sister emerges^1^, appear more easily misled by orthology errors than site-heterogeneous approaches^15^, with which we observe a shift towards support for Porifera-sister in R&M^1^. Lastly, we disagree that discussing W&H’s simulations^16^ comparing the Phylobayes^17^ CAT^18^ model with partitioning is irrelevant, as although we did not directly use the CAT model, we did employ partitioning with previously defined, precomputed variants of CAT^19^ using IQ-tree^20^ in R&M^1^.

Our points above refute W&H’s arguments that our use of recoding was spurious and we strongly reject the notion that we do not accurately present results favouring Ctenophora-sister (branch and partition-specific support values were fully reported in R&M^1^) and do not apply an ‘objective lens’ in our interpretations^2^. Rather W&H’s assertion that ‘partitioning with linked branches and site-heterogeneous models recovered the Ctenophora-sister hypothesis’^2^ disregards the clear pattern observed in R&M^1^ for all datasets of increasing support for Porifera-sister (over Ctenophora-sister) as model fit increases, downplaying this as reduced support in ‘some’ datasets/analyses^2^. In summary, our primary conclusions remain firmly intact.

## Methods

### Unpartitioned, site-heterogeneous, SR4-recoded animal phylogenomics

SR4^21^ recoded phylogenomic analyses were performed on the REA, WEA15, and WEA17 datasets from R&M^1^ using IQ-tree version 1.6.12^20^ and employing 1000 ultrafast bootstrap replicates^22^. All analyses performed here were unpartitioned in order to test whether poor resolution and inconsistent results between datasets are ascribable to (i) SR4 recoding (as contended by W&H^2^) or (ii) simple, flat F81 exchangeabilities (as proposed here and in R&M), which were always better-fitting than GTR exchangeabilities when combining partitioning, recoding and site-heterogeneous models in R&M^1^. The logic behind this is that when a single model is applied to the entire phylogenomic dataset rather than separate models to each partition, then there may be enough data for site-heterogeneous models with GTR, rather than simpler F81, exchangeabilities to fit best when recoding is applied. ModelFinder^23^ in IQ-tree was used to assess the best-fitting models under both the AIC and BIC. The site-homogeneous models F81 and GTR were tested, as well as pairings of each of these exchangeability matrices with SR4 recoded derivations (from R&M^1^) of the site-heterogenous C10, C20, C30, C40, C50, and C60 precomputed CAT models^19^. All site-heterogeneous models also incorporated 4 discrete gamma categories to help accommodate rate heterogeneity across sites. For example, the model ‘SR4C60GTR’ (see naming as applied in Fig. 1a), is SR4 recoded, employs GTR exchangeabilities, the 60 site frequency categories from C60, and 4 discrete gamma categories for rate heterogeneity. Each dataset was analysed under the best fitting GTR-based model (GTR-based models always fit best under AIC), as well as under the best-fitting F81-based model (best-fitting under BIC for REA and WEA15), enabling comparison of the resultant maximum likelihood consensus trees and support values between GTR-based and F81-based analyses, as well as with previous F81-based partitioned analyses performed in R&M^1^.

### Ancestral Ctenophora branch length as an LBA severity measure

The long ancestral ctenophore branch (i.e., the internal branch leading to the extant ctenophores in the animal tree of life) has been suggested to cause LBA between Ctenophora and non-animal outgroups, producing tree topologies supporting Ctenophora as sister to all other animals^1,7,8,10,12^. We contend that the longer this branch is (i.e., the more substitutions per site along this branch) in a given dataset (branch length may vary due to substitution model applied, differing gene and site content, variation in alignment and orthology errors, etc.) the more difficult it will be to overcome potential LBA of Ctenophora towards the root of the animal tree when analysing that dataset. We extracted this branch length value from the maximum likelihood consensus trees resulting from standard partitioned phylogenomics of the REA, WEA15, and WEA17 datasets performed in R&M (i.e., named analysis level ‘L1’ in R&M^1^). Branch lengths were plotted in Fig. 1b for comparison across datasets, both in ‘Raw’ form (as directly extracted from the consensus trees) and in ‘Normalised’ form (raw branch length divided by total tree length for that dataset, then multiplied by the average total tree length of the three datasets).

### Reporting summary

Further information on research design is available in the Nature Portfolio Reporting Summary linked to this article.

## Supplementary information

Reporting Summary

## Data Availability

Datasets and tree files from our reanalyses are available at 10.6084/m9.figshare.16856152. Derivations of the C10-C60 precomputed CAT models for SR4 recoding are from R&M^1^ and available at 10.6084/m9.figshare.12746972.
