# Supplementary material for: Reply to: Available data do not rule out Ctenophora as the sister group to all other Metazoa
Source: Nat Commun. 2023 Feb 10;14:710. doi: 10.1038/s41467-023-36152-5 (PMC9918546; doi:10.1038/s41467-023-36152-5)
Supplement: Supplementary file 1 — Reporting Summary [file 41467_2023_36152_MOESM1_ESM.pdf]

nature portfolio

Corresponding author(s): Anthony K Redmond, Aoife McLysaght

Last updated by author(s): Jan 16, 2023

Reporting Summary

Nature Portfolio wishes to improve the reproducibility of the work that we publish. This form provides structure for consistency and transparency in reporting. For further information on Nature Portfolio policies, see our Editorial Policies and the Editorial Policy Checklist.

Statistics

For all statistical analyses, confirm that the following items are present in the figure legend, table legend, main text, or Methods section.

n/a	Confirmed	
☒	☐	The exact sample size (*n*) for each experimental group/condition, given as a discrete number and unit of measurement
☒	☐	A statement on whether measurements were taken from distinct samples or whether the same sample was measured repeatedly
☒	☐	The statistical test(s) used AND whether they are one- or two-sided<br>*Only common tests should be described solely by name; describe more complex techniques in the Methods section.*
☒	☐	A description of all covariates tested
☒	☐	A description of any assumptions or corrections, such as tests of normality and adjustment for multiple comparisons
☒	☐	A full description of the statistical parameters including central tendency (e.g. means) or other basic estimates (e.g. regression coefficient) AND variation (e.g. standard deviation) or associated estimates of uncertainty (e.g. confidence intervals)
☒	☐	For null hypothesis testing, the test statistic (e.g. *F*, *t*, *r*) with confidence intervals, effect sizes, degrees of freedom and *P* value noted<br>*Give P values as exact values whenever suitable.*
☒	☐	For Bayesian analysis, information on the choice of priors and Markov chain Monte Carlo settings
☒	☐	For hierarchical and complex designs, identification of the appropriate level for tests and full reporting of outcomes
☒	☐	Estimates of effect sizes (e.g. Cohen's *d*, Pearson's *r*), indicating how they were calculated

Our web collection on statistics for biologists contains articles on many of the points above.

Software and code

Policy information about availability of computer code

Data collection	No specific software or software packages were used for dataset collection. Datasets were reanalysed from the original Redmond and McLysaght (2021) study.
Data analysis	IQ-tree version 1.6.12 (obtained from http://www.iqtree.org/) was used to infer the new phylogenetic trees presented in figure 1a.

For manuscripts utilizing custom algorithms or software that are central to the research but not yet described in published literature, software must be made available to editors and reviewers. We strongly encourage code deposition in a community repository (e.g. GitHub). See the Nature Portfolio guidelines for submitting code & software for further information.

Data

Policy information about availability of data

All manuscripts must include a data availability statement. This statement should provide the following information, where applicable:

- Accession codes, unique identifiers, or web links for publicly available datasets
- A description of any restrictions on data availability
- For clinical datasets or third party data, please ensure that the statement adheres to our policy

Datasets and tree files from our reanalyses are available at https://doi.org/10.6084/m9.figshare.16856152. Derivations of the C10-C60 precomputed CAT models for SR4 recoding are from R&M1 and available at https://doi.org/10.6084/m9.figshare.12746972.

Human research participants

Policy information about studies involving human research participants and Sex and Gender in Research.

Reporting on sex and gender	N/A
Population characteristics	N/A
Recruitment	N/A
Ethics oversight	N/A

Note that full information on the approval of the study protocol must also be provided in the manuscript.

Field-specific reporting

Please select the one below that is the best fit for your research. If you are not sure, read the appropriate sections before making your selection.

☐ Life sciences ☐ Behavioural & social sciences ☒ Ecological, evolutionary & environmental sciences

For a reference copy of the document with all sections, see nature.com/documents/nr-reporting-summary-flat.pdf

Ecological, evolutionary & environmental sciences study design

All studies must disclose on these points even when the disclosure is negative.

Study description	The study is a reply to matters arising piece and includes reanalyses of existing phylogenomic datasets.
Research sample	The research samples are animal phylogenomic datasets that are derived from other studies and were previously complied and reanalysed in our original study that this piece derives from (Redmond and McLysaght, 2021).
Sampling strategy	The current study is restricted to reanalysis of pre-existing datasets.
Data collection	Data were obtained directly from previous studies - the key datasets and models used here were obtained from Redmond and McLysaght (2021).
Timing and spatial scale	N/A - The current study is restricted to reanalysis of pre-existing datasets
Data exclusions	No data were excluded from reanalyses performed here as compared to Redmond and McLysaght (2021). However we note that 1) We employed an amino acid recoding strategy that places amino acids into bins that essentially mask some amino acid changes (such recoding has been applied widely but arguments in the matters arising and this reply centre around this procedure), and 2) We discarded gene partition information from our phylogenetic analyses in this manuscript as compared to Redmond and McLysaght to disentangle to effects of recoding from the effects of model choice induced by partitioning phylogenomic datasets.
Reproducibility	Our analyses are readily reproducible when tested using IQ-tree.
Randomization	N/A
Blinding	No blinding was performed during data analyses - which consisted entirely of reanalyses of datasets analysed in previous studies.

Did the study involve field work? ☐ Yes ☒ No

Reporting for specific materials, systems and methods

We require information from authors about some types of materials, experimental systems and methods used in many studies. Here, indicate whether each material, system or method listed is relevant to your study. If you are not sure if a list item applies to your research, read the appropriate section before selecting a response.

Materials & experimental systems

n/a	Involved in the study
☒	☐ Antibodies
☒	☐ Eukaryotic cell lines
☒	☐ Palaeontology and archaeology
☒	☐ Animals and other organisms
☒	☐ Clinical data
☒	☐ Dual use research of concern

Methods

n/a	Involved in the study
☒	☐ ChIP-seq
☒	☐ Flow cytometry
☒	☐ MRI-based neuroimaging
